# Supplementary material for: Kdm1a safeguards the topological boundaries of PRC2-repressed genes and prevents aging-related euchromatinization in neurons
Source: Nat Commun. 2024 Mar 7;15:1781. doi: 10.1038/s41467-024-45773-3 (PMC10920760; doi:10.1038/s41467-024-45773-3)
Supplement: Supplementary file 1 — Supplementary Information [file 41467_2024_45773_MOESM1_ESM.pdf]

Kdm1a safeguards the topological boundaries of PRC2-repressed genes and prevents aging-related euchromatinization in neurons

del Blanco el al.

Supplementary materials

- Supplementary Figures 1 to 9 with legends
- Supplementary Table 1
- Supplementary References

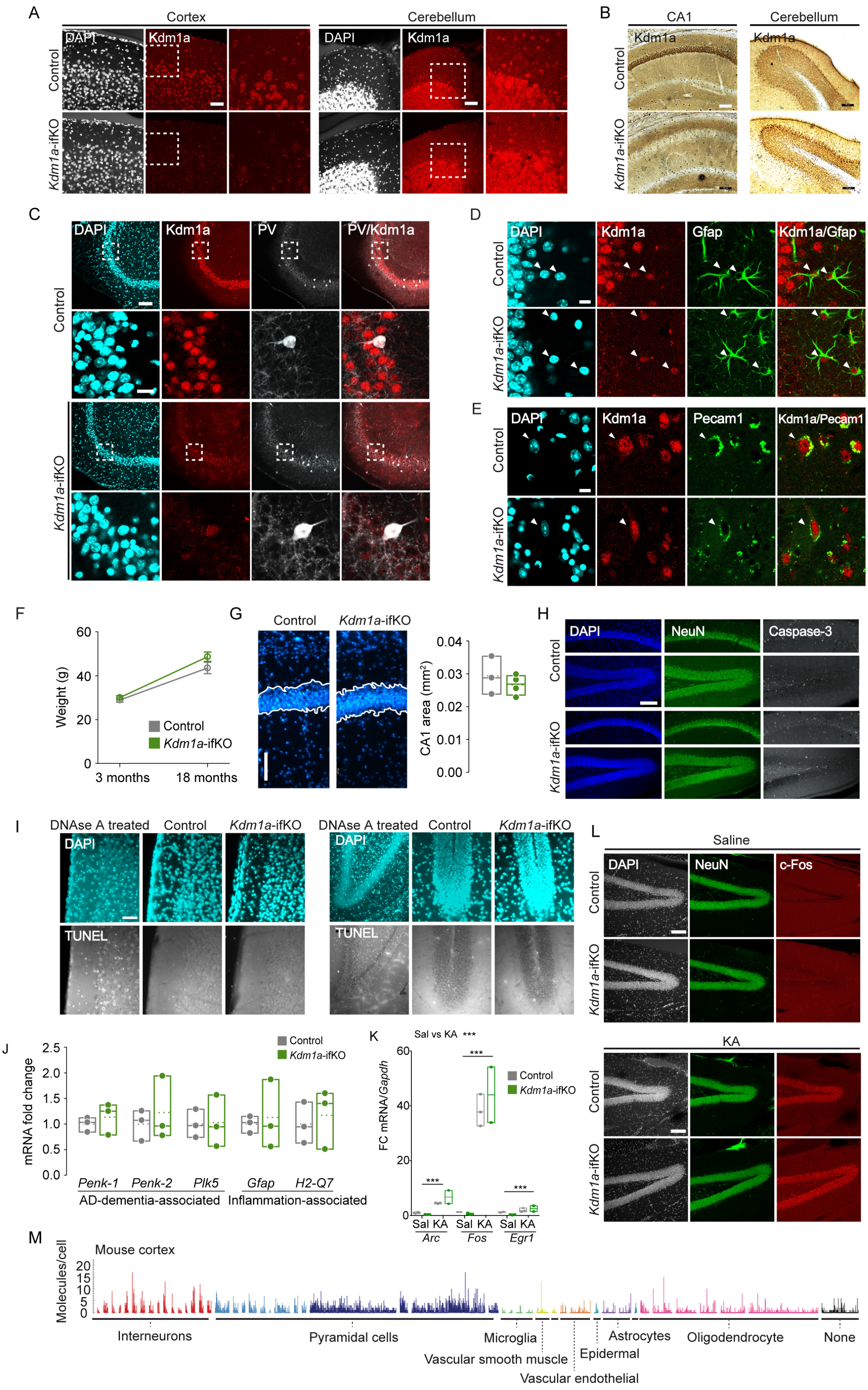
A
Cortex
Cerebellum
DAPI
Kdm1a
Control
Kdm1a-ifKO
B
CA1
Cerebellum
Kdm1a
C
DAPI
Kdm1a
PV
PV/Kdm1a
D
Gfap
Kdm1a/Gfap
E
Pecam1
Kdm1a/Pecam1
F
Weight (g)
3 months
18 months
G
CA1 area (mm²)
H
NeuN
Caspase-3
I
DNAse A treated
TUNEL
L
Saline
c-Fos
KA
J
mRNA fold change
Penk-1
Penk-2
Plk5
Gfap
H2-Q7
AD-dementia-associated
Inflammation-associated
K
Sal vs KA ***
FC mRNA/Gapdh
Sal
Arc
Fos
Egr1
M
Mouse cortex
Molecules/cell
Interneurons
Pyramidal cells
Microglia
Vascular smooth muscle
Vascular endothelial
Epidermal
Astrocytes
Oligodendrocyte
None

Supplementary Figure 1. Forebrain-specific ablation of Kdm1a in principal neurons. A-B. Immunofluorescence (A) and DAB immunostaining (B) using anti-Kdm1a antibody confirm the depletion of Kdm1a in cortical and hippocampal neurons of Kdm1a-ifKOs. Kdm1a is not eliminated in cerebellum and other brain areas in which the *Camk2a* driver is not active. Scale bar: 50 µm (A), 100 µm (B). **C.** Co-immunostaining using α-Kdm1a and α-parvalbumin (PV) shows the expression of Kdm1a in interneurons. Scale bar: 100 µm and 20 µm (insets). **D-E.** Co-immunostaining using α-Kdm1a and α-GFAP (D) or α-Pecam1 (E) confirms the expression of Kdm1a in astrocytes and endothelial cells. Arrowheads highlight representative cells. Scale bar: 10 µm. **F.** Body weight of mice across their lifespan (3 months: n = 13 for CT, n = 12 for Kdm1a-ifKO; $t_{23} = -0.816$, $p = 0.423$; 18 months: n = 12 for CT, n = 11 for Kdm1a-ifKO; $t_{21} = -1.512$, $p = 0.145$, t-test). **G.** DAPI staining and quantification of the thickness of the CA1 area in Kdm1a-ifKO and control littermates (n = 3 for WT, n = 4 for Kdm1a-ifKO; T = 14, p = 0.629, Mann-Whitney Rank Sum test). Scale bar 50 µM. **H.** Staining for active Caspase-3 demonstrates that CA1 and dentate gyrus hippocampal neurons in *Kdm1a*-ifKOs do not display apoptosis. Scale bar: 100 µm. **I.** TUNEL cell death assay in the cortex and hippocampus of WT and *Kdm1a*-ifKO mice. DNAse A-treated slices from control mice are included as a control. Scale bar: 100 µm. **J.** RT-qPCR assay for genes associated with cell death and neuro-inflammation did not show significant differences between *Kdm1a*-ifKOs and control littermates. **K.** RT-PCR analysis of mRNA levels for three representative IEGs (*Arc, Fos* and *Egr1*) in the hippocampus of Kdm1a-ifKOs and control littermates. mRNA samples were extracted 1 h after treatment with kainic acid (KA). We observed a significant effect of treatment (***: $p > 0.001$) but no significant effect of genotype. **L.** NeuN and Fos immunostaining of the dentate gyrus from 18-month-old Kdm1a-ifKO and control littermates (16 months after TMX) administered with saline or KA 1 hour before sacrificing. Scale bar: 100 µm **M.** *Kdm1a* expression in different cell types according to single-cell transcriptome data in the mouse cortex generated by the Linnarsson's lab [1].

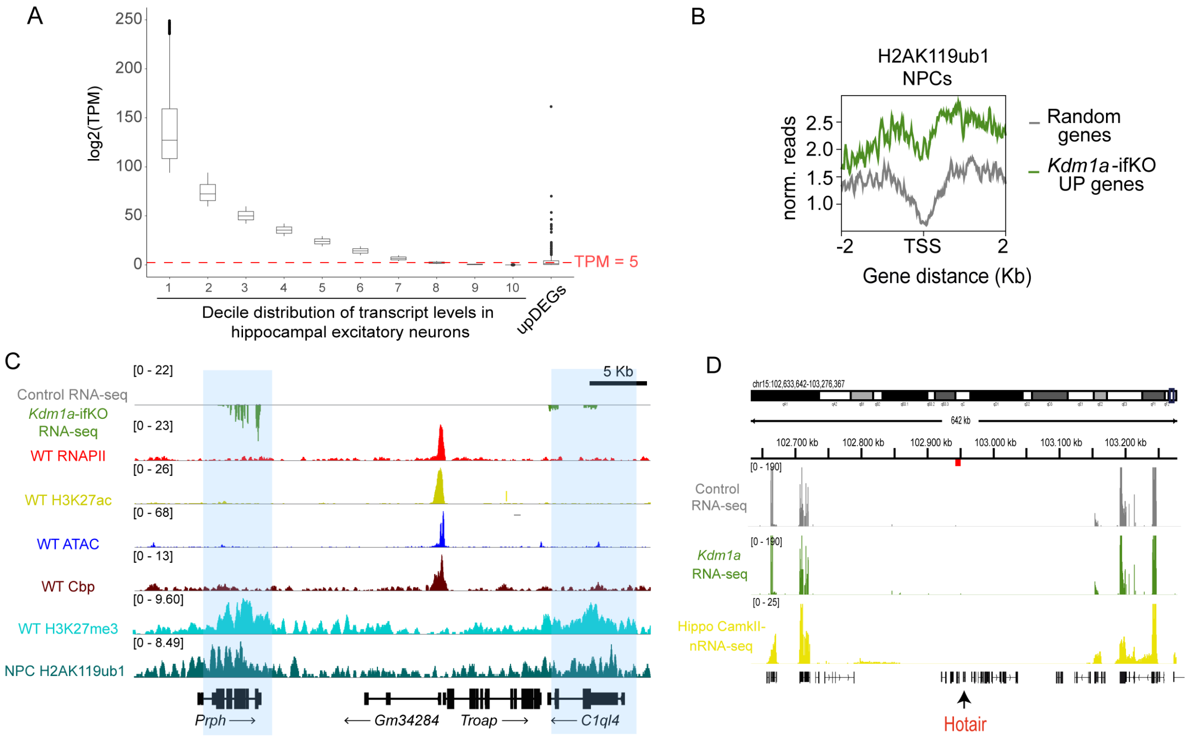

Supplementary Figure 2. Kdm1a loss causes de-repression of PRC2-repressed nonneuronal genes A. Box plot comparing the expression level of upDEGs with those of other genes The series of decile ranks show the absolute expression value (in transcripts per million, TPM) of all coding transcripts detected in excitatory neurons of the adult mouse hippocampus [2]. The first decile box does not show the 248 genes with values above 250 TPMs. **B.** H2AK119ub1 enrichment in the chromatin of WT NPCs at the TSS (-2 to +2 Kb) of Kdm1a-ifKO upregulated compared to a similar number of randomly selected genes expressed in neurons. **C.** Genomic snapshot shows a representative example of two upregulated genes in Kdm1a-ifKOs, *Prph* and *C1ql4*, which are in close proximity to each other. Data range is shown in brackets. Arrows indicate the sense of transcription. All genomic profiles correspond to mouse hippocampus except for H2AK119ub1. **D.** Profile of *Hotair* expression comparing nuclear RNA from excitatory neurons and bulk hippocampal Kdm1a-ifKO and control samples of adult mouse brain

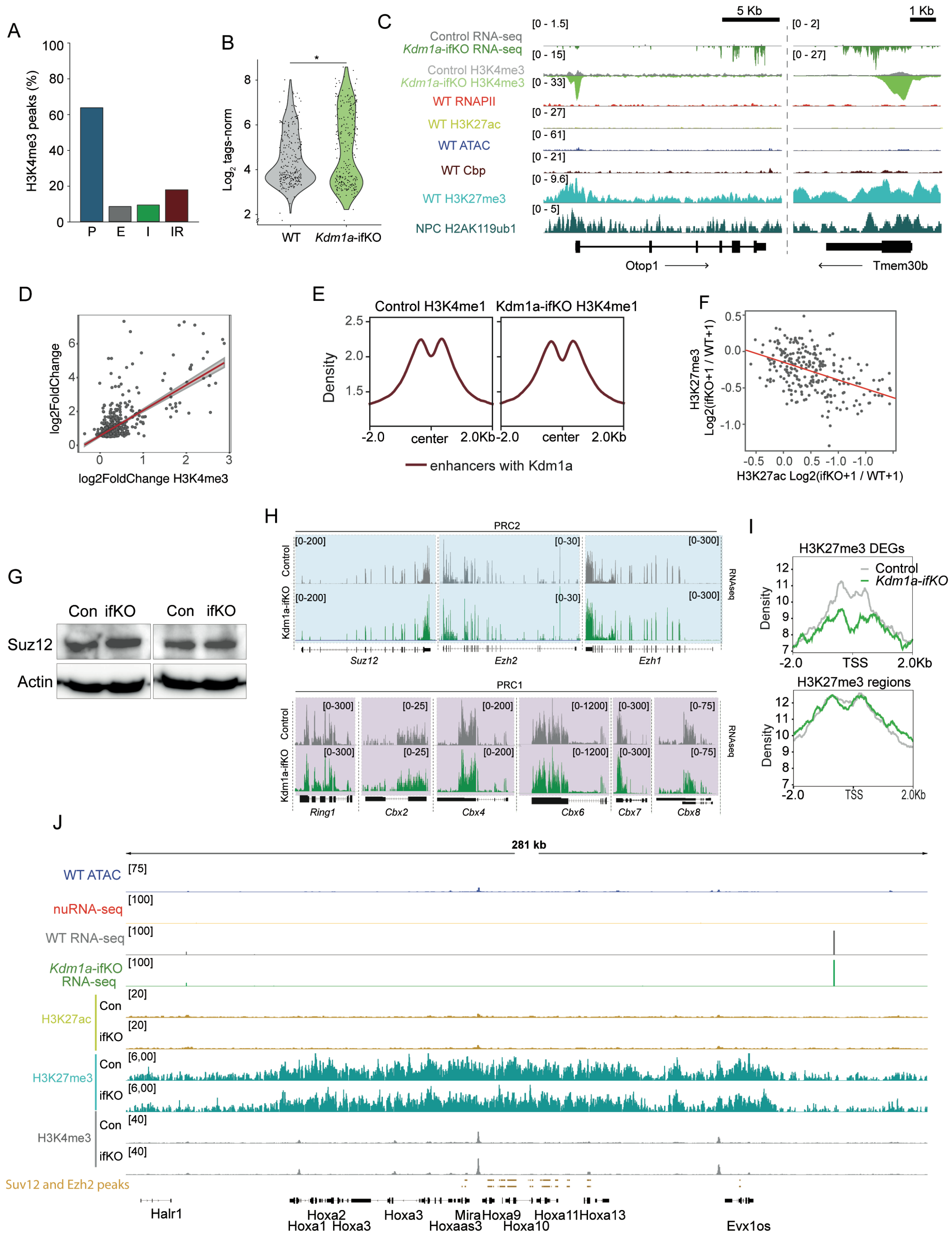
A
H3K4me3 peaks (%)
P
E
I
IR
B
Log2 tags-norm
WT
Kdm1a-ifKO
C
Control RNA-seq
Kdm1a-ifKO RNA-seq
Control H3K4me3
Kdm1a-ifKO H3K4me3
WT RNAPII
WT H3K27ac
WT ATAC
WT Cbp
WT H3K27me3
NPC H2AK119ub1
5 Kb
1 Kb
Otop1
Tmem30b
D
log2FoldChange
log2FoldChange H3K4me3
E
Control H3K4me1
Kdm1a-ifKO H3K4me1
Density
center
enhancers with Kdm1a
F
H3K27me3 Log2(ifKO+1 / WT+1)
H3K27ac Log2(ifKO+1 / WT+1)
G
Con ifKO
Con ifKO
Suz12
Actin
H
PRC2
Control
Kdm1a-ifKO
RNAseq
Suz12
Ezh2
Ezh1
PRC1
Ring1
Cbx2
Cbx4
Cbx6
Cbx7
Cbx8
I
H3K27me3 DEGs
Control
Kdm1a-ifKO
Density
TSS
H3K27me3 regions
J
281 kb
WT ATAC
nuRNA-seq
WT RNA-seq
Kdm1a-ifKO RNA-seq
H3K27ac
Con
ifKO
H3K27me3
H3K4me3
Suv12 and Ezh2 peaks
Halr1
Hoxa2
Hoxa1
Hoxa3
Hoxa3
Hoxaas3
Mira
Hoxa9
Hoxa10
Hoxa11
Hoxa13
Evx1os

Supplementary Figure 3. Dramatic changes in epigenetic profiles of Kdm1a-ifKOs. A. Bar plot of the distribution of H3K4me3 DMRs in Kdm1a-ifKO mice. P = promoters; E = exons; I = introns; IR = intergenic regions. **B**. Violin plot shows that Kdm1a-ifKO upregulated genes display higher H3K4me3 levels in Kdm1a-ifKO mice than their control littermates ($p < 0.05$, Mann-Whitney Rank Sum test). **C**. Genomic snapshot of representative genes upregulated in Kdm1a-ifKO mice that exhibit an increment in H3K4me3 at their promoter regions, which are depleted of active genomic marks and display H3K27me3 and H2AK119ub1. Data range is shown in brackets. Arrows indicate the sense of transcription. All genomic profiles correspond to mouse hippocampus except for H2AK119ub1. **D.** Correlation between changes in H3K4me3 levels (log2FoldChange) and changes in upDEG transcript levels (log2FoldChange). Multiple lineal regression analysis: p value = $3.02e\text{-}^{24}$. **E.** Density plot of H3K4me1 signal in hippocampal chromatin of Kdm1a-ifKOs and control littermates at enhancer regions sorted by Kdm1a signal. **F.** Negative correlation between changes in H3K27me3 and H3K27ac in hippocampus of Kdm1a-ifKO compared with control littermate mice. **G.** Western blot of hippocampal protein extract shows no changes in the Suz12 PCR2 subunit protein level in Kdm1a-ifKO mice compared to control mice. Actin protein level as a control. **H.** Genomic snapshot indicating similar RNA levels of the major subunits of PRC2 (*Suz12, Ezh1,* and *Ezh2*) and PRC1 (*Ring1 Cbx2, Cbx4, Cbx6, Cbx7,* and *Cbx8*) from hippocampal RNA-seq samples from Kdm1a-ifKO and control mice. **I.** Changes in H3K27me3 levels in the chromatin of Kdm1a-ifKO and control littermates. To demonstrate the specificity of the effect we compared the set of upDEGs (upper graph) versus all genes displaying H3K27me3-high enrichment (lower graph). **J.** Genomic snapshot of representative the epigenetic profile of the *Hoxa* gene cluster in chromosome 6. Epigenetic profiles used: ATAC-seq and nuRNA-seq of mouse hippocampus excitatory neurons, RNA-seq; H3K27ac, H3K27me3 and H3K4me3 from hippocampus of Kdm1a-ifKO and control mice. The orange bars indicate the genomic coordinates of the peaks found in ChIP-seq of the PRC2 subunits Suz12 and Ezh2 in the chromatin of neural progenitors.

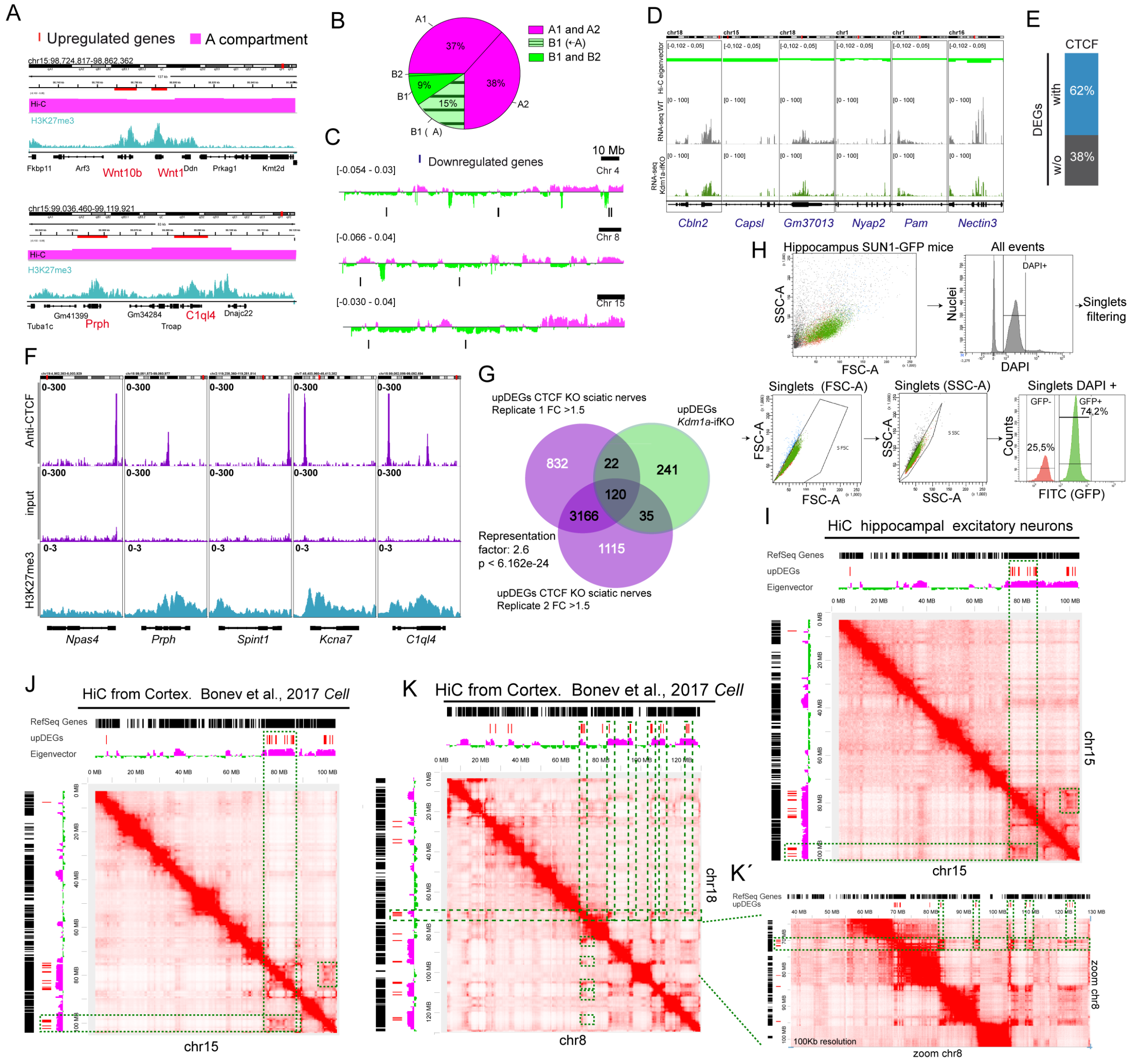

Supplementary Figure 4. upDEGs cluster in silent stretches of euchromatin. A. Zoomed snapshots showing the clustering of the upDEGs *Wnt10b, Wnt1, Prph* and *C1ql4*, their enrichment in H3K27me3 and location into compartment A. **B.** Kdm1a-regulated genes are distributed between the sub-compartments A1 and A2 in adult excitatory neurons with no clear preference for one sub-compartment. This analysis also revealed that 15% of the upDEGs that mapped into the A compartment at 25Kb resolution now mapped into the B1 compartment defined in the 250Kb map (see Methods). The result suggests that these genes locate near the boundary between the A and B compartments. **C.** Genomic snapshots of the A/B compartments in chromosomes 4, 8, and 15 indicating the location of genes downregulated (in blue) in *Kdm1a*-ifKO mice. **D.** Zoomed genomic snapshots showing some genes downregulated in Kdm1a-ifKO mice that are located in compartment B. **E.** Percentage of Kdm1a-ifKO upregulated genes with CTCF peak (5 kb down TSS and / or 1 kb up TSS gene) from the CTCF Chip-seq previously published by Shen Y *et al.* [3]. **F.** Snap view of CTCF binding and H3K27me3 signal at the promoter of Kdm1a-repressed genes. *Npas4* promoter is shown as a representative CTCF-bound neuronal gene. **G.** Overlap of upregulated genes (FC ≥ 1.5) in two replicates of CTCF KO mouse sciatic nerves published by Wang *et al.* [4] and Kdm1a upregulated gene (FC ≥ 0.5). The overlap between *bona fide* upregulated genes in CTCF (3,286 genes) and upDEGs is larger than expected by chance (representation factor 2.6 p < 6.162e-24). **H.** Channel filtering of flow cytometry signal for the isolation of singlet nuclei of hippocampal excitatory neurons for ChIA-PET. The last panel shows the green fluorescence signal in Sun1-GFP$^-$ and Sun1-GFP$^+$ nuclei. **I-K.** Hi-C contact matrices of excitatory neurons from adult hippocampus (**I**) [2] and embryonic cortex (**J-K**) [5] visualized with Juicebox software. Intrachromosomal interactions of Chr5 and Chr8 are used as representative examples of the interactions of upregulated genes (red bars) in Kdm1a-ifKO mice.

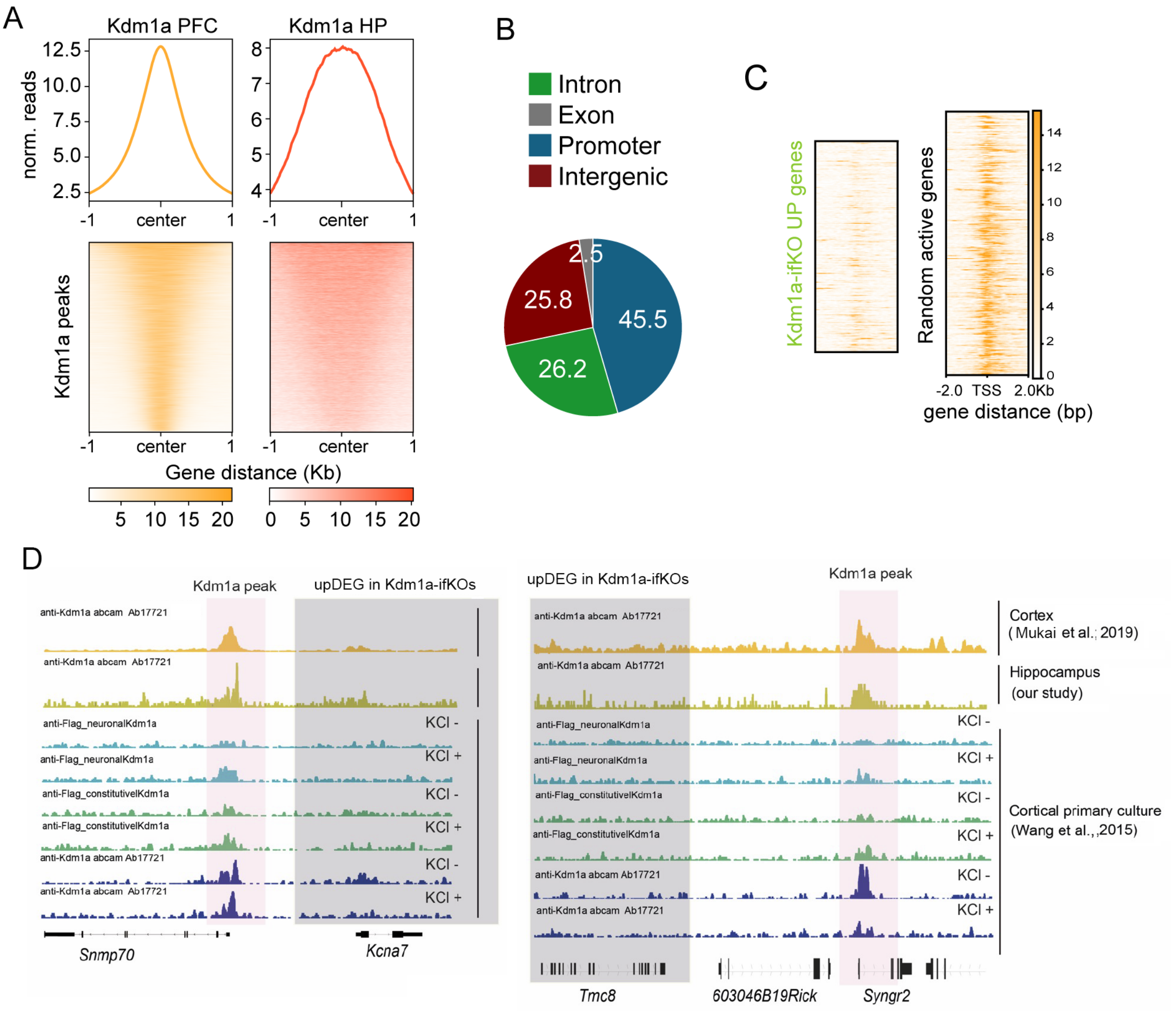

Supplementary Figure 5. upDEGs are brough together by CTCF loops. A. Graphs and their corresponding heatmaps evidence the similarity between Kdm1a ChIP-seq datasets from adult mouse prefrontal cortex (PFC) and hippocampus (HP). **B.** Pie Charts shows the distribution of Kdm1a peaks in the PFC of adult mice. **C.** Heatmap shows Kdm1a enrichment in TSS of upregulated genes compared to TSS of transcriptionally active genes in neurons**. D.** Genomic snapshots encompassing two upregulated differentially expressed genes (upDEGs): *Tmc8* and *Kcna7*. The comparison of the Kdm1a ChIP-seq profiles generated in three independent studies revealed enrichment in the same regions. In alignment with our findings (this study), the profiles from cortex (Mukai et al., 2019) and neuronal cultures (Wang et al., 2015) also revealed a weak binding of Kdm1a to the upDEGs.

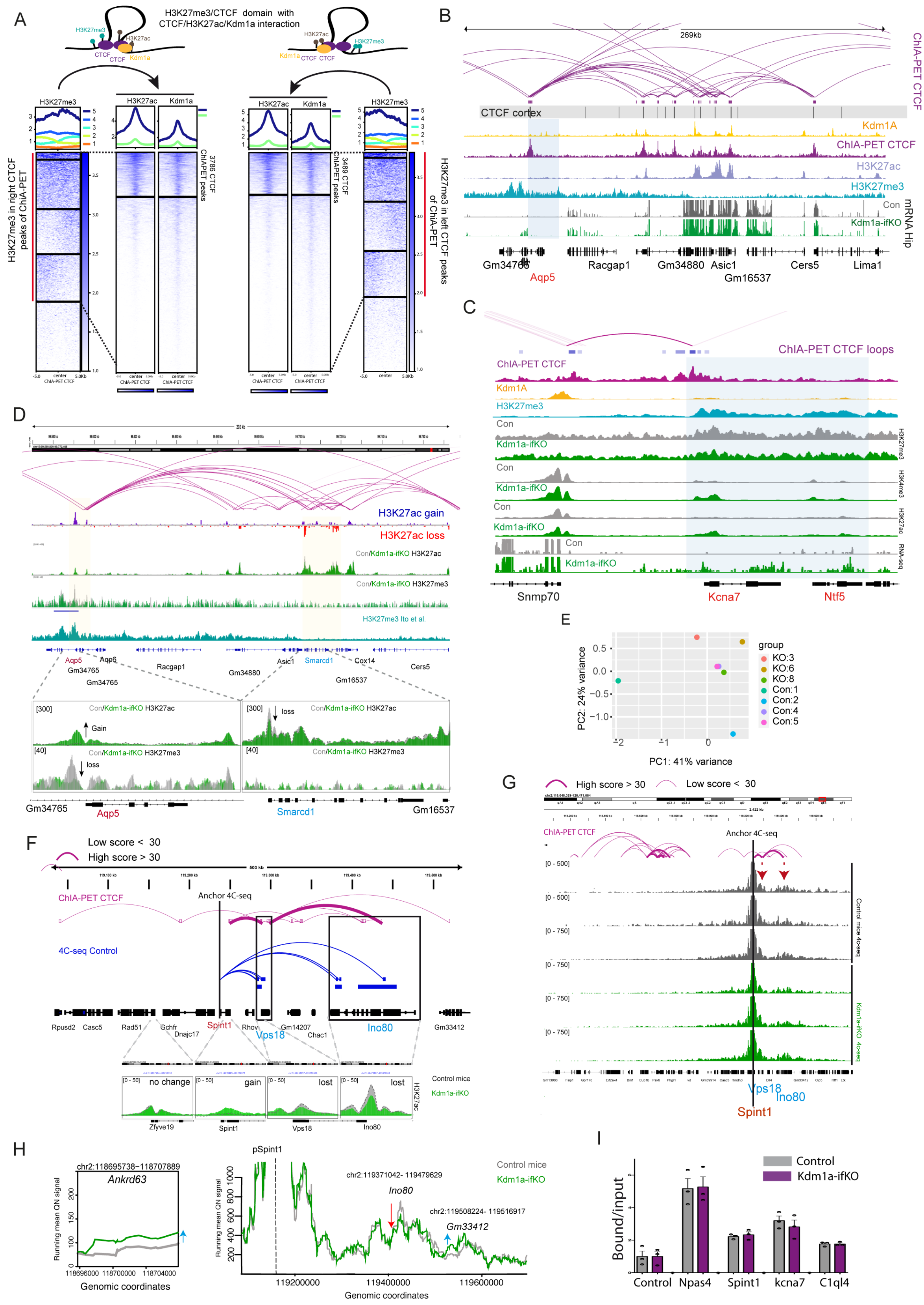
A
H3K27me3/CTCF domain with CTCF/H3K27ac/Kdm1a interaction
H3K27me3 in right CTCF peaks of ChIA-PET
3786 CTCF ChIAPET peaks
3489 CTCF ChIAPET peaks
H3K27me3 in left CTCF peaks of ChIA-PET
B
269kb
ChIA-PET CTCF
CTCF cortex
Kdm1A
H3K27ac
H3K27me3
Con
Kdm1a-ifKO
mRNA Hip
Gm34766
Aqp5
Racgap1
Gm34880
Asic1
Gm16537
Cers5
Lima1
C
ChIA-PET CTCF loops
Snmp70
Kcna7
Ntf5
D
H3K27ac gain
H3K27ac loss
Con/Kdm1a-ifKO H3K27ac
Con/Kdm1a-ifKO H3K27me3
H3K27me3 Ito et al.
Gain
loss
Gm34765
Smarcd1
E
PC2: 24% variance
PC1: 41% variance
group
KO:3
KO:6
KO:8
Con:1
Con:2
Con:4
Con:5
F
Low score < 30
High score > 30
Anchor 4C-seq
4C-seq Control
Spint1
Vps18
Ino80
no change
gain
lost
Control mice
G
High score > 30
Low score < 30
Control mice 4c-seq
Kdm1a-ifKO 4c-seq
H
chr2:118695738-118707889
Ankrd63
Running mean ON signal
Genomic coordinates
pSpint1
chr2:119371042- 119479629
chr2:119508224- 119516917
Gm33412
I
Bound/input
Control
Kdm1a-ifKO
Npas4
kcna7
C1ql4

Supplementary Figure 6. CTCF loops approach Kdm1a to its targets. A. Schemes show the configuration of CTCF loops displaying an enrichment for H3K27me3 in either one or both sides (aprox. 70% of the total number of loops). Density plot for H3K27me3 in the CTCF-loops. Four H3K27me3-enriched clusters are observed. A subset of H3K27me3-rich regions interacts with regions enriched in Kdm1a and H3K27ac (3,786 regions, 20% of CTCF-loops enriched in H3K27me3) via CTCF-looping. **B-C.** Genomic snapshots of two representative upDEGs enriched in H3K27me3 (*Kcna7* and *Aqp5,* in red). CTCF-ChIA-PET revealed the topological association of Kdm1a with *Aqp5* (**B**) and *Kcna7* (**C**). CTCF binding is indicated by a blue square. Purple lines indicate CTCF-mediated interactions. **D.** Genomic profiles and CTCF ChIA-PET interactions at the representative upDEG *Aqp5* locus (in red). Note the topological association with regions enriched in H3K27ac. CTCF loops bring the H3K27me3-enriched gene *Aqp5* closer to *Smarcd1*, which is transcribed and enriched in H3K27ac. The bottom panels show the comparison of H3K27ac genomic profiles at *Aqp5* and *Smarcd1* in control and Kdm1a-ifKOs. **E.** Principal Component Analysis (PCA) of 4C-seq samples. **F.** 4C-seq interaction profiles using *Spint1* promoter as a viewpoint (anchor). Note the coincidence with CTCF-dependent interactions observed in the ChIA-PET of hippocampus excitatory neurons. *Vps18* and *Ino80* loci are label in red. **G.** Genomic snapshots of 4C-seq data in control mice (blue) confirm the CTCF-dependent interactions detected by ChIA-PET between the *Spint1* promoter and *Vps18* and *Ino80*. The comparison of H3K27ac profiles at *Spint1*, *Vps18* and *Ino80* in control and Kdm1a-ifKOs is shown on the bottom panels. **H.** Genomic Snapshot of 4C profiles generated with the 4See browser. These profiles represent the average normalized interaction frequency of the *Spint1* promoter with the genes *Ankrd65, Ino80* and *Gm33412*, that undergo significant changes in 4C-seq interactions between Kdm1a-ifKOs and control littermates. **I.** qPCR-ChIP analysis of three representative upregulated genes in Kdm1a-ifKOs: *Spint1*, *Kcna7* and *C1ql4* mice demonstrates no change in CTCF binding to the sites identified published by Shen and colleagues [3].

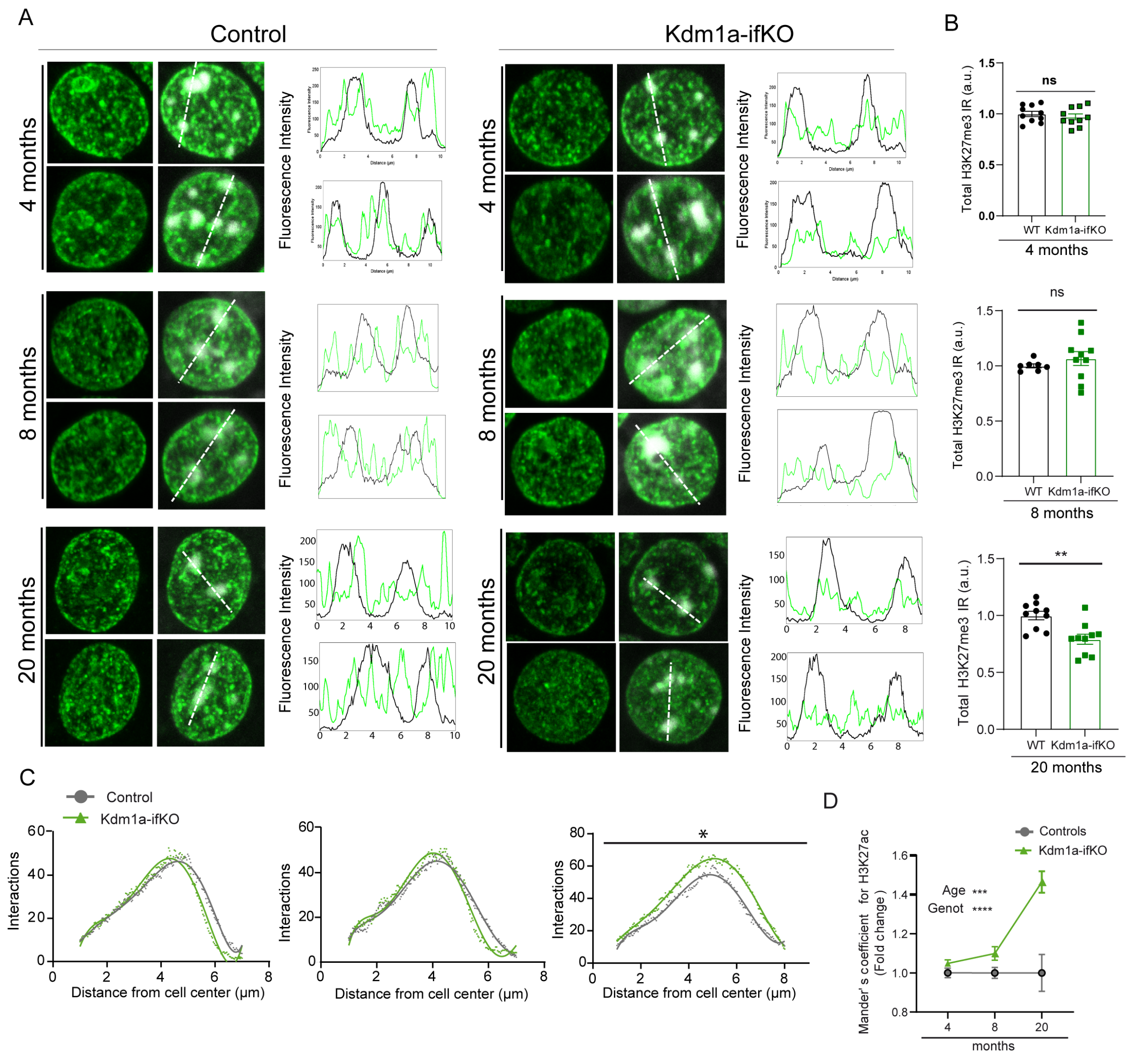

Supplementary Figure 7. Analysis of the H3K27me3 signal and its progression with age. A. Representative Z-project immunofluorescence confocal images reflect the H3K27me3 nuclear pattern in hippocampal neurons of Kdm1a-ifKO mice compared to control mice neurons. The dashed white line indicates the area used in the line scan analysis generated with the ImageJ software analysis program. Line-scan profiles of fluorescence intensity confirm an altered pattern of fluorescence intensity and distribution of H3K27me3 in the nuclei of Kdm1a-ifKO neurons compared with control mice. The black line of the diagram indicates the fluorescence intensity of the DAPI and the green line the fluorescence intensity of the H3K27me3 signal. **B.** Measurement of H3K27me3 signal in the nucleus of CA1 hippocampal neurons of 4-, 8- and 20-month-old Kdm1a-ifKO and control littermates. **C.** Sholl analysis of the H3K27me3 binary signal demonstrates a global change in nuclear distribution in 20-month-old Kdm1a mice compared to controls. **D.** H3K27ac and H3K27me3 colocalization was measured using Mander's coefficient.

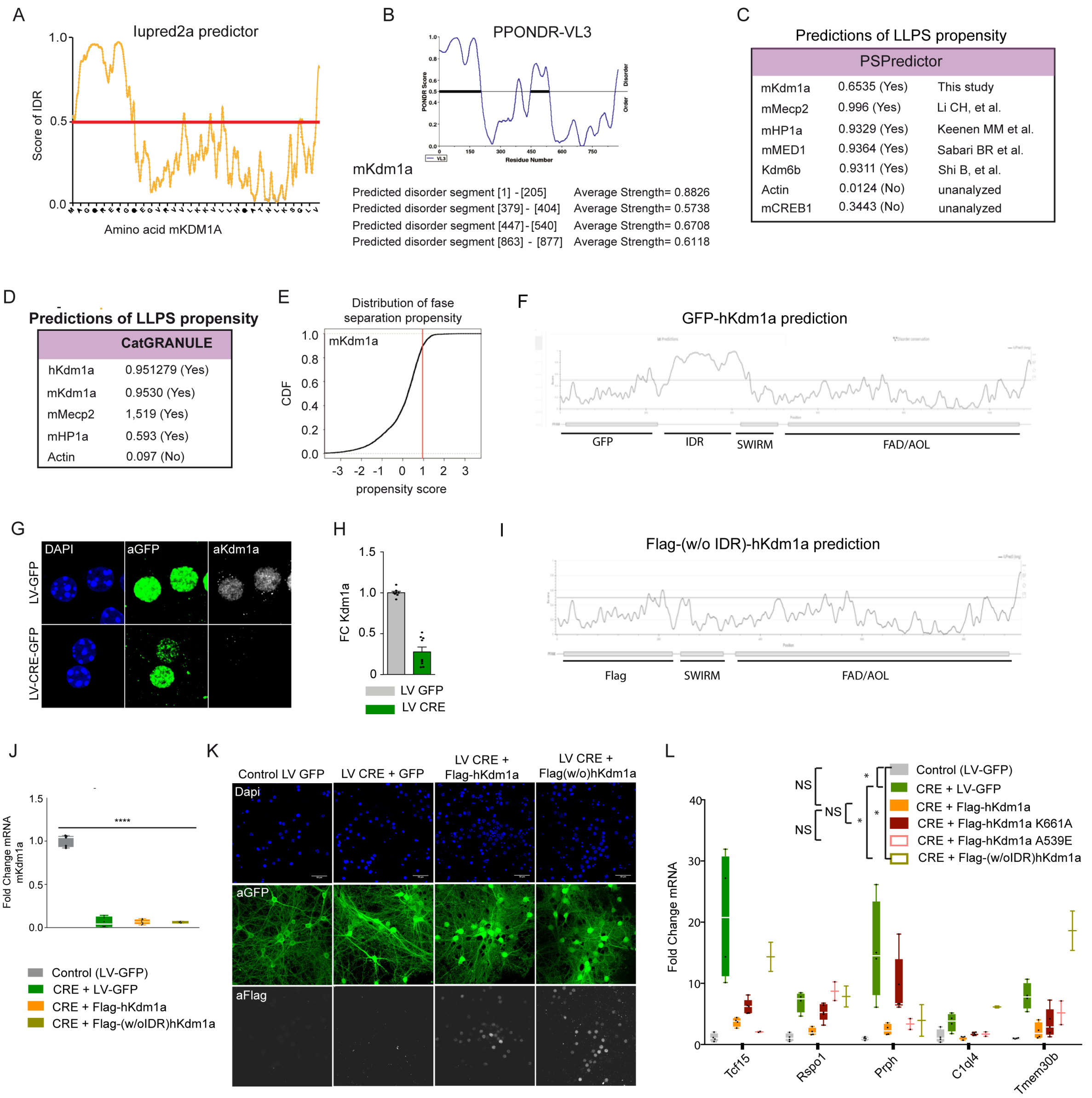

Supplementary Figure 8. Kdm1a is involved in LLPS. A. Intrinsically disordered region prediction in the mouse Kdm1a protein using IUPred2A [6]. **B.** Intrinsically disordered regions prediction using PONDR-VL3 [7] This algorithm detected a long IDR in the N-terminal region and a shorter IDR in the center region. **C.** PSPredictor indicates that Kdm1a may have LLPS properties. Its score is similar to those of proteins with known LLPS properties. As a negative control, we also show the scores for beta-actin and the TF CREB. **D-E.** Human and mouse Kdm1a score similarly to well-characterized PSPs, such as Mecp2 and HP1a, using the CatGRANULE algorithm. **F.** IDR prediction for GFP-hKdm1a using IUPred2A. **G.** Immunolabelling of hippocampal PNCs infected with GFP-expressing or GFP and Cre recombinase expressing lentiviruses were stained with anti-Kdm1a antibody. **H.** RT-qPCR assay demonstrates the reduced expression of Kdm1a 12 days after infection with LV-CRE-GFP in PNCs. **I.** IDR prediction for Flag-(w/o IDR)-hKdm1a using IUPred2A. In this truncated protein the algorithm does not predict any IDR. **J.** RT-qPCR assay demonstrates the loss of endogenous Kdm1a transcripts in PNCs infected with control LV-GFP or co-infected with LV-CRE plus control LV-GFP; or LV-CRE plus LV-Flag-hKdm1a; or LV-CRE plus LV-Flag (w/oIDR)-hKdm1a**.** Note that hKdm1a transcripts are not recognized with the primer pair used to quantify mKdm1a transcripts. **K.** Immunostainings using anti-Flag antibody demonstrate the presence of Flag-hKdm1a and Flag-(w/o-IDR) hKdm1a in PNCs infected with the indicated viruses. **L.** RT-qPCR assays demonstrate that human Kdm1a bearing mutations that affect H3K4me2 demethylase catalytic activity (K661A and K598E) largely prevented the activation of upDEGs, although less efficiently than hKdm1a. *: p-value < 0.05 in multiple comparison test after one-way ANOVA.

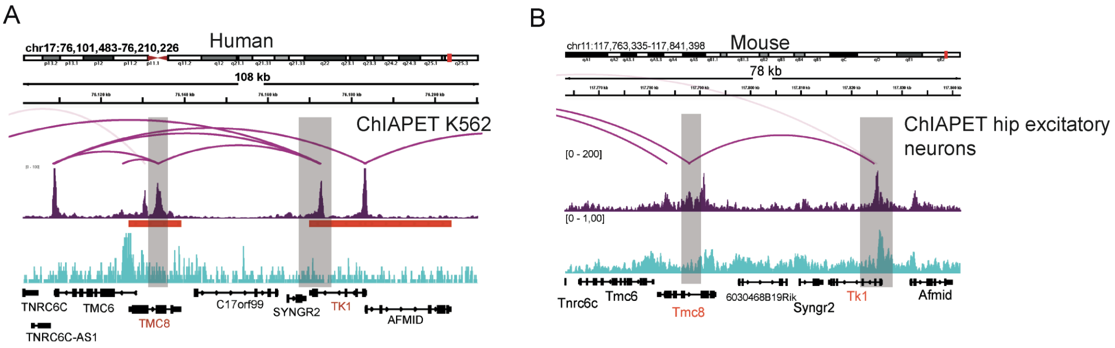

Supplementary Figure 9. Spatial synteny of human and mouse chromatin structure in TMC8-TK1 cluster genes. A-B Genomic snapshot of the interaction between the genes upregulated in Kdm1a-deficient neurons *Tmc8* and *Tk1* (**A**) and their human homologs (**B**), which are also upregulated during aging. The comparison of human CTCF ChIA-PET data [8] and the CTCF ChIA-PET data generated in mouse hippocampal excitatory neurons demonstrates the conservation of the 3D chromatin interaction between TMC8 and TK1, which could explain their co-upregulation during aging.

Supplementary Table 1. Primer sequences

RT-qPCR primers pairs

**Target**	**Forward**	**Reverse**
Kdm1a	ATACTGTGCTTGTCCACCGAG	[illegible]TAGCTGTCGAGCTG
Penk1	GGCGTGCACACTGGAATGT	TCCCAGATTTTGAAAGAAGGCA
Penk2	CCTCAAAAGAAGCCCCCAAC	CGCTTCTGCAGCTCTTTTGC
Plk5	CACCTGTGTTTGCCTTTCCC	TTTTGAGGTAAAGGGACAAGGTG
Gfap	GGACAACTTTGCACAGGACCTC	TCCAAATCCACACGAGCCA
H2-Q7	AGGAGCAGAATTACACATGCCA	CGCCATGTTGGAGACAGTGTAT
C1ql4	TCGACCTCACACTTCTTGCC	CAGCCGCGGAATCCCTAATA
Prph	TCGATTTCTCCATGGCCGAG	CGGTCGTTGAGCTCCTGTAA
Spint1	TGCACCACTCAGAACTGCAA	GAGCGAACTTGCACACGAAG
Tcf15	TCCATTCGGTTATGCCTCTC	CACCTGGCAGTCTTCAGAGTC
Ltbp2	GATGGAGATGTGGCAAAAAGA	GTGGAGCAAGAGAGGGTTTG
Bmp8a	GAGGCAGGAAACCCTTCTATG	AGGACAGAGGAACTGGGAAA
Mixl1	TCCCTGAGTGCATCCTCAAC	CGTCTGCACATCTGGTTCTC
Rspo1	GTGGATTCAAGCAGGACAGG	CTGGGTCAGTGGCAGGAG
Tcf24	AACGCTCCAGCCTCTAGATTG	ACCCAGTTAAGGTAGGCGAC
Kcna7	CTTCGATGCGGTGCTCTACT	CAGCCCGTAGAAGGACACC
Tmem30b	GCATGACAAAGCCCATGACG	GGCACCTACCGTGTCAACAT

ChIP-qPCR primers pairs

**Target**	**Forward**	**Reverse**
C1ql4	TCGACCTCACACTTCTTGCC	CAGCCGCGGAATCCCTAATA
Tmem30b	GCATGACAAAGCCCATGACG	GGCACCTACCGTGTCAACAT
Prph	TCGATTTCTCCATGGCCGAG	CGGTCGTTGAGCTCCTGTAA
HPCA	GCATGCGTAATCAGTTCTGGA	CTGCACAGGAAGGAGTCAGA
Kcna7	TGGTACACATCTGTCATCCCA	GCCTCTGTATCTCTCTGCCT
Spint1	CAGGGGTTAATGCCAGTCCC	CTCTGGCTGCACGGAAGATT
Spint1 CTCF	GGTGACTGGCTAGAGGAGGA	ACCGAGAGCAGAGATCCAGA
Kcna7 CTCF	CGAGATCCCTTTCCCCGAAA	GATGCGTAGGGTCACTGTCG
C1ql4 CTCF	TCTCAGCCCTCACCGTATGT	ACCCCTGTTTACCCCATCAC
Npas4 CTCF	CTGCCTCGATCCGCCTT	GTCTCTAAAAACGAGCCCCC

hKdm1a IDR depletion

Forward	GACAGTCCTCGAGTTCCAGAGCCGA CTTCCTCATG	GGTTGATTGCGGCCGCTCA

4C-seq primer pair

4C Reading p1 Spint1	ACTGGAGTTCAGACGTGTGCTCTTCCGATCTCCGGAAGTTCTGCTGGGC
non_blind p1 Spint1	TACACGACGCTCTTCCGATCTATAGGCTGCGTTTGTGTAAAC

Supplementary References

1. Zeisel, A. *et al.* Brain structure. Cell types in the mouse cortex and hippocampus revealed by single-cell RNA-seq. *Science (80-.).* **347**, 1138–1142 (2015).
2. Fernandez-Albert, J. *et al.* Immediate and deferred epigenomic signatures of in vivo neuronal activation in mouse hippocampus. *Nat Neurosci* **22**, 1718–1730 (2019).
3. Shen, Y. *et al.* A map of the cis-regulatory sequences in the mouse genome. *Nature* **488**, 116–120 (2012).
4. Wang, J. *et al.* CTCF-mediated chromatin looping in EGR2 regulation and SUZ12 recruitment critical for peripheral myelination and repair. *Nat. Commun.* **11**, 4133 (2020).
5. Bonev, B. *et al.* Multiscale 3D Genome Rewiring during Mouse Neural Development. *Cell* **171**, 557-572 e24 (2017).
6. Mészáros, B., Erdős, G. & Dosztányi, Z. IUPred2A: context-dependent prediction of protein disorder as a function of redox state and protein binding. *Nucleic Acids Res.* **46**, W329–W337 (2018).
7. Peng, K. *et al.* Optimizing long intrinsic disorder predictors with protein evolutionary information. *J. Bioinform. Comput. Biol.* **03**, 35–60 (2005).
8. Fullwood, M. J., Han, Y., Wei, C., Ruan, X. & Ruan, Y. Chromatin Interaction Analysis Using Paired-End Tag Sequencing. *Curr. Protoc. Mol. Biol.* **89**, (2010).
