# Supplementary material for: Kdm1a safeguards the topological boundaries of PRC2-repressed genes and prevents aging-related euchromatinization in neurons
Source: Nat Commun. 2024 Mar 7;15:1781. doi: 10.1038/s41467-024-45773-3 (PMC10920760; doi:10.1038/s41467-024-45773-3)
Supplement: Supplementary file 3 — Description of Additional Supplementary Files [file 41467_2024_45773_MOESM3_ESM.pdf]

Description of Additional Supplementary Files

File Name: Supplementary Data 1
Description: Differentially expressed genes in Kdm1a-ifKO neurons of the adult mouse brain.

File Name: Supplementary Data 2
Description: CTCF ChIA-PET interactions with a score ≥ 3.

File Name: Supplementary Data 3
Description: Statistic of 4C-seq experiment.

File Name: Supplementary Data 4
Description: Kdm1a-dependent genes that exhibit age-dependent expression changes in the human brain.
