# Supplementary material for: Kdm1a safeguards the topological boundaries of PRC2-repressed genes and prevents aging-related euchromatinization in neurons
Source: Nat Commun. 2024 Mar 7;15:1781. doi: 10.1038/s41467-024-45773-3 (PMC10920760; doi:10.1038/s41467-024-45773-3)
Supplement: Supplementary file 8 — Reporting Summary [file 41467_2024_45773_MOESM8_ESM.pdf]

nature portfolio

Corresponding author(s): Angel Barco

Last updated by author(s): Jan 17, 2024

Reporting Summary

Nature Portfolio wishes to improve the reproducibility of the work that we publish. This form provides structure for consistency and transparency in reporting. For further information on Nature Portfolio policies, see our Editorial Policies and the Editorial Policy Checklist.

Statistics

For all statistical analyses, confirm that the following items are present in the figure legend, table legend, main text, or Methods section.

n/a	Confirmed	
☐	☒	The exact sample size (*n*) for each experimental group/condition, given as a discrete number and unit of measurement
☐	☒	A statement on whether measurements were taken from distinct samples or whether the same sample was measured repeatedly
☐	☒	The statistical test(s) used AND whether they are one- or two-sided<br>*Only common tests should be described solely by name; describe more complex techniques in the Methods section.*
☒	☐	A description of all covariates tested
☐	☒	A description of any assumptions or corrections, such as tests of normality and adjustment for multiple comparisons
☐	☒	A full description of the statistical parameters including central tendency (e.g. means) or other basic estimates (e.g. regression coefficient) AND variation (e.g. standard deviation) or associated estimates of uncertainty (e.g. confidence intervals)
☐	☒	For null hypothesis testing, the test statistic (e.g. *F*, *t*, *r*) with confidence intervals, effect sizes, degrees of freedom and *P* value noted<br>*Give P values as exact values whenever suitable.*
☒	☐	For Bayesian analysis, information on the choice of priors and Markov chain Monte Carlo settings
☒	☐	For hierarchical and complex designs, identification of the appropriate level for tests and full reporting of outcomes
☒	☐	Estimates of effect sizes (e.g. Cohen's *d*, Pearson's *r*), indicating how they were calculated

Our web collection on statistics for biologists contains articles on many of the points above.

Software and code

Policy information about availability of computer code

Data collection	Equipment software of Applied Biosystems 7300 real-time PCR unit, FACS Aria III, Illumina HiSeq2500, Leica stereo microscope, Olympus confocal microscope, Confocal Microscope Zeiss LSM 880-Airyscan Elyra PS.1.
Data analysis	General sotware: Microsoft Excel 14.7.3; Adobe Photoshop and Adobe Illustrator CS6.<br><br>Statistics: R Studio v3.5.1; GraphPad Prism v8.0.1.<br><br>Image analysis: Fiji – ImageJ 2.0.0-rc-68, ZEN 2.3 software, Huygens Professional version 21.10 and Imaris 8.2.0<br><br>Genomic analysis: Cutadapt v1.18; Samtools v1.9; Bedtools v2.27.1; DeepTools v3.5.0; IGV v2.5.0; DESeq2 v1.18.1; HISAT2 v2.1.0; TrimGalore v 0.6.4_dev; Rsubread v. 2.4.3; HTseq v0.11.1; GOstats v2.44.0; Picardtools v2.18.21; Bowtie2 v2.3.4.2; MACS2 v2.1.1; DiffBind v2.10.0; ChIPpeakAnno v3.24.2; Biomart v2.46.3; homer v4.11.1; ggplot2 v3.3.3; FastQC v0.11.9. ChIA-PIPE pipeline.<br><br>We used the following publicly available webtools: Enrichr (https://maayanlab.cloud/Enrichr/), Gene List Venndiagram (https://www.bioinformatics.org/gvenn), PAVIs annotation peaks (https://manticore.niehs.nih.gov/pavis2). Juicebox: https://www.aidenlab.org/juicebox/

For manuscripts utilizing custom algorithms or software that are central to the research but not yet described in published literature, software must be made available to editors and reviewers. We strongly encourage code deposition in a community repository (e.g. GitHub). See the Nature Portfolio guidelines for submitting code & software for further information.

Data

Policy information about availability of data

All manuscripts must include a data availability statement. This statement should provide the following information, where applicable:

- Accession codes, unique identifiers, or web links for publicly available datasets
- A description of any restrictions on data availability
- For clinical datasets or third party data, please ensure that the statement adheres to our policy

The genomic data sets generated in this study can be accessed at the GEO public repository using the accession number GSE236182. In addition, we used several previously published datasets: GSE123652: Kdm1a ChIP-seq from adult PFC; GSE133018: CBP and H3K27ac ChIP-seq from adult mouse hippocampus; GSE56810: H3K27me3 ChIP-seq from adult mouse hippocampus; GSE125068: RNAPII ChIP-seq from adult mouse hippocampus, and Hi-C, ATAC-seq and nuclear RNA-seq from sorted mouse hippocampal principal neurons; GSM2393589: H2AK119ub1 ChIP-seq from mouse NPCs; GSM1479208: H2AK119ub1 ChIP-seq from mouse brai; GSM918727: CTCF ChIP-seq from adult mouse cortex; GSM1917302: Suz12, and GSM1917302: Ezh2 ChIP-seq from NPCs. Sub-compartment information come from Supplementary Data 5 in Chandrasekaran et al., 2021, which applies k-based sub-compartment mapping to the neuron-specific Hi–C data from adult mouse hippocampus was used in the analyses presented in Fig. 3C and Fig. 4B. Human RNA expression data from control postmortem brain samples were obtained from GSE33000, GSE15222 and GSE48350.

Research involving human participants, their data, or biological material

Policy information about studies with human participants or human data. See also policy information about sex, gender (identity/presentation), and sexual orientation and race, ethnicity and racism.

Reporting on sex and gender	N/A
Reporting on race, ethnicity, or other socially relevant groupings	N/A
Population characteristics	N/A
Recruitment	N/A
Ethics oversight	N/A

Note that full information on the approval of the study protocol must also be provided in the manuscript.

Field-specific reporting

Please select the one below that is the best fit for your research. If you are not sure, read the appropriate sections before making your selection.

☒ Life sciences ☐ Behavioural & social sciences ☐ Ecological, evolutionary & environmental sciences

For a reference copy of the document with all sections, see nature.com/documents/nr-reporting-summary-flat.pdf

Life sciences study design

All studies must disclose on these points even when the disclosure is negative.

Sample size	Sample size was determined according to data variance and correlation between biological replicates and distance to control condition. Samples sizes were sufficient according to statistical methods, confidence intervals and p-values. Each analysis was done in accordance to the sample size, sequencing depth and conditions.
Data exclusions	Sample WT1 in the 4C-seq experiment was considered an outlier and was not included in subsequent statistical analyses according to the initial PCA and visualization. Two samples corresponding to the ChIP-seq experiment for H3K4me1 (one per genotype) were considered outliers and were excluded in subsequent statistical analyses according to the PCA result and visualization in the Genome browser.
Replication	All replication attempts of the experiments were successful.<br>The measures applied to evaluate replicates were correlation among samples, variance, Euclidean classification, principal component analysis (PCA) and statistical tests revealed non significant differences within the group. In the analyses in which a replicate was not available, analyses were supported by the use of internal controls and the sample was normalized and compared with samples from related conditions in which replicates were available (i.e., ANOVA, DESeq2).<br>All histological experiments (IHC, TUNEL) were replicated at least twice or had at least three biological replicates. All the molecular experiments (ChIP, mRNA), with the exception of ChiAPET-CTCF, were replicated twice and validated by qPCR. All experiments in primary hippocampus cultures were replicated at least three times in different cultures.
Randomization	Set of mice with the same age and sex were raised in the same conditions and randomly allocated to the different experimental groups. Researchers were blinded to group allocation during data collection. For experiments other than those involving mice, experimental design did not involve randomization.

Blinding	Researchers were blinded to group allocation during data collection in all the experiments in which the subjective view of the experimenter could influence the result. Investigators were not blinded to group allocation during bioinformatical, molecular and cellular biology analyses. These analyses followed pre-determined pipelines and blinding was not relevant statistical analysis.

Reporting for specific materials, systems and methods

We require information from authors about some types of materials, experimental systems and methods used in many studies. Here, indicate whether each material, system or method listed is relevant to your study. If you are not sure if a list item applies to your research, read the appropriate section before selecting a response.

Materials & experimental systems

n/a	Involved in the study
☐	☒ Antibodies
☐	☒ Eukaryotic cell lines
☒	☐ Palaeontology and archaeology
☐	☒ Animals and other organisms
☒	☐ Clinical data
☒	☐ Dual use research of concern
☒	☐ Plants

Methods

n/a	Involved in the study
☐	☒ ChIP-seq
☐	☒ Flow cytometry
☒	☐ MRI-based neuroimaging

Antibodies

Antibodies used

The following primary antibodies have been used in this study:

Immunohistochemistry:
α-Kdm1a (ab17721, 1:200 or 1:1000)
α-NeuN (Chemicon MAB377, 1:500)
α-GFAP (G3893, Sigma, 1:500)
α-Cleaved Caspase-3 (9661, Cell Signalling, 1:500)
α-Fos (226004, Synaptic Systems, 1:500)
α-Parvalbumin (P3088, Sigma-Aldrich, 1:500)
α-Pecam 1 (550274, BD Pharmingen™, 1:500)
α-H3K27me3 (07-449, Millipore, 1:500)
α-H3K27me3 (ab6002, Abcam, 1:250)
α-H3K27ac (ab4729, Abcam, 1:500).

Western blot:
α-SUZ12 antibody (ab175187 Abcam, 1:500)
α-β-actin monoclonal antibody (clone AC-74, Merck, 1:500)

ChIA-PET:
α-CTCF-specific antibody (Abclonal Cat # ab70303)

ChIP antibodies:
α-Kdm1a (ab17721, Abcam)
α-H3K4me3 (07-473, Millipore)
α-H3K4me1 (ab8895, Abcam)
α-H3K27ac (ab4729, Abcam),
α-CTCF (07-729, Millipore)
α-H3K27me3, (07-449, Millipore)

Validation

All the commercial antibodies are validated by the manufacturer and/or scientific publications. We detail the links to the manufacturers' websites that contain validation for the techniques used.

Immunohistochemistry:
α-Kdm1a (ab17721, Abcam)
https://www.abcam.com/products/primary-antibodies/kdm1lsd1-antibody-nuclear-marker-ab17721.html

α-NeuN (Chemicon MAB377, 1:500)
https://www.merckmillipore.com/ES/es/product/Anti-NeuN-Antibody-clone-A60,MM_NF-MAB377

α-GFAP (G3893, Sigma, 1:500)
https://www.sigmaaldrich.com/ES/en/product/sigma/g3893

α-Cleaved Caspase-3 (9661, Cell Signalling, 1:500)
https://www.cellsignal.com/products/primary-antibodies/cleaved-caspase-3-asp175-antibody/9661

α-Fos (226004, Synaptic Systems, 1:500)

https://sysy.com/product/226004

α-Parvalbumin (P3088, Sigma-Aldrich, 1:500)
https://www.sigmaaldrich.com/ES/en/product/sigma/zrb1218?gclid=EAIaIQobChMI4vS578T9ggMVhYVoCR0C1wwsEAAYASAAEgLIP_D_BwE

α-Pecam 1 (550274, BD Pharmingen™, 1:500)
https://www.scbt.com/p/pecam-1-antibody-mec-13-3?gad_source=1&gclid=EAIaIQobChMIiPyAh8X9ggMVhopoCR2hCwplEAAYASAAEgI2xfD_BwE

α-H3K27ac (ab4729, Abcam)
https://www.abcam.com/products/primary-antibodies/histone-h3-acetyl-k27-antibody-chip-grade-ab4729.html

α-H3K27me3 (ab6002, Abcam, 1:250)
https://www.abcam.com/products/primary-antibodies/histone-h3-tri-methyl-k27-antibody-mabcam-6002-chip-grade-ab6002.html

α-H3K27me3, (07-449, Millipore)
https://www.merckmillipore.com/ES/es/product/Anti-trimethyl-Histone-H3-Lys27-Antibody,MM_NF-07-449

Western blot:
α-SUZ12 antibody (ab175187 Abcam, 1:500)
https://www.abcam.com/products/primary-antibodies/suz12-antibody-epr5234n-chip-grade-ab175187.html

α-β-actin monoclonal antibody (clone AC-74, Merck, 1:500)
https://www.sigmaaldrich.com/ES/es/product/sigma/a5316

ChIA-PET:
α-CTCF
Abclonal Cat # ab70303 was used as described in the in situ ChIA-PET protocol (Wang et al., 2021)
https://www.abcam.com/products/primary-antibodies/ctcf-antibody-ab70303.html

ChIPs:
α-Kdm1a (ab17721, Abcam)
https://www.abcam.com/products/primary-antibodies/kdm1lsd1-antibody-nuclear-marker-ab17721.html

α-H3K4me3 (07-473, Millipore)
https://www.merckmillipore.com/ES/es/product/Anti-trimethyl-Histone-H3-Lys4-Antibody,MM_NF-07-473

α-H3K4me1 (ab8895, Abcam)
https://www.abcam.com/products/primary-antibodies/histone-h3-mono-methyl-k4-antibody-chip-grade-ab8895.html

α-H3K27ac (ab4729, Abcam)
https://www.abcam.com/products/primary-antibodies/histone-h3-acetyl-k27-antibody-chip-grade-ab4729.html

α-CTCF (07-729, Millipore)
https://www.merckmillipore.com/ES/es/product/Anti-CTCF-Antibody,MM_NF-07-729

α-H3K27me3, (07-449, Millipore)
https://www.merckmillipore.com/ES/es/product/Anti-trimethyl-Histone-H3-Lys27-Antibody,MM_NF-07-449

Eukaryotic cell lines

Policy information about cell lines and Sex and Gender in Research

Cell line source(s)	Human Embryonic Kidney (HEK) 293 (CRL-3216 ™ from ATTC)
Authentication	The line has not been authenticated.
Mycoplasma contamination	The cell line was tested negative for mycoplasma contamination.
Commonly misidentified lines (See ICLAC register)	No commonly misidentified cell lines were used

Animals and other research organisms

Policy information about studies involving animals; ARRIVE guidelines recommended for reporting animal research, and Sex and Gender in Research

Laboratory animals	Mouse strains were maintained in a pure C57BL/6J background. Kdm1af/f (Kerenyi et al., 2013), CAG-Sun1/sfGFP (Mo et al., 2015) and Camk2a-creERT2 (Erdmann et al., 2007) mice have been previously described and are available at public depositories (Jackson Lab stock #23969, MMRRC stock 066789-UCD; Jackson Lab stock #021039, RRID:IMSR_JAX:021039 and EMMA EM:02125, respectively). All mice were maintained on a C57BL/6J genetic background.<br>Mice were maintained and bred under standard conditions, consistent with Spanish and European regulations. All the protocols for animal experimentation were approved by the Animal Welfare Committee at the Instituto de Neurociencias, the CSIC Ethical

Committee and the Dirección General de Agricultura, Ganadería y Pesca of Generalitat Valenciana. In detail, mice were maintained under specific pathogen-free (SPF) conditions within the Animal House at the Instituto de Neurociencias (CSIC-UMH), in a 12 h light/12 h dark cycle (7:00 a.m. to 7:00 p.m.) at 20-24ºC and controlled humidity (40-60%) with free access to food and water. Kdm1af/f mice bear loxP sites flanking exons 5 and 6; their recombination results in the appearance of a premature stop codon in the mRNA. Camk2a-creERT2 x Kdm1af/f mice selectively eliminate Kdm1a in principal neurons of the forebrain after TMX administration in 8-week-old mice. Camk2a-creERT2 x CAG-Sun1/sfGFP were used to isolate principal neurons nuclei of the forebrain in 12-week-old mice after one month of TMX administration. For phenotypic analyses, young adult (3 month-old, 1-month after TMX), adult (9 month-old, 7 months after TMX) and aged (18 month-old, 16 months after TMX) mice were used as indicated.
Primary hippocampal and cortical cultures were prepared from Kdm1af/f from 3-month old pregnant mothers with the same genotypes.

Wild animals: The study does not involve the use of wild animals.

Reporting on sex: Most of the experiments are generated with male mice for a greater homogeneity and minimize the number of animals used per experiment The 4C-seq, ChiA-PET-CTCF, RNA-seq and ChiP-seq are data generated with male mice. CTCF qChiP RT-PCR was generated with female. Super-resolution images n=12 male and n= 6 female mice). Primary hippocampal cultures were generated from a mix of male and female embryos.

Field-collected samples: The study does not involve field-collected samples

Ethics oversight: Animals were housed according to the Spanish and European regulations and the experiments were approved by the Animal Welfare Committee at the Instituto de Neurociencias, the CSIC Ethical Committee and the Dirección General de Agricultura, Ganadería y Pesca of Generalitat Valenciana.

Note that full information on the approval of the study protocol must also be provided in the manuscript.

Plants

Seed stocks: N/A

Novel plant genotypes: N/A

Authentication: N/A

ChIP-seq

Data deposition

☒ Confirm that both raw and final processed data have been deposited in a public database such as GEO.

☒ Confirm that you have deposited or provided access to graph files (e.g. BED files) for the called peaks.

Data access links
May remain private before publication.

https://www.ncbi.nlm.nih.gov/geo/query/acc.cgi?acc=GSE236182

Files in database submission

KDM1a_merge.bam.RPKM.bw, KDM1a_wt_merge.bam.RPKM.bw
S1K_merged_mapq30.bam.bw, S1W_merged_mapq30.bam.bw,
S3K_merged.mapq30.bam.SCALESIZEFACTORS.bw,S3W_merged.mapq30.bam.SCALESIZEFACTORS.bw,WTLSD1_H3K27ac.mapq30.bam.RPGC.bw,WTLSD1_H3K27me3.mapq30.bam.SCALESIZEFACTORS.bw,
KDM1a_allmerged_hippocampus.bam.RPGC.bw
ChIA-PET_mm10_Hippocampus_ctrl_CTCF_LMH0019_0019H_0019N_hiseq_pairs.hic,
LMH0019_0019H_0019N.e500.clusters.cis.BE3.G50.bothpksupport.bedpe, LMH0019_0019H_0019N.for.BROWSER.bigwig,
LMH0019_0019H_0019N.for.BROWSER.sorted.bedgraph
1WT_spint_WIN21.wig, 2WT_spint_WIN21.wig, 3KO_spint_WIN21.wig, 4WT_spint_WIN21.wig, 4WT_spint_WIN21.wig,
6KO_spint_WIN21.wig, 8KO_spint_WIN21.wig

Raw files:
W45_31144_ATCACG.fastq.gz, W47_31145_CGATGT.fastq.gz, W48_31146_TTAGGC.fastq.gz, K46_31147_TGACCA.fastq.gz,
K50_31148_ACAGTG.fastq.gz, K52_31149_GCCAAT.fastq.gz, W45_31144_ATCACG_2.fastq.gz,
W47_31145_CGATGT_2.fastq.gz, W48_31146_TTAGGC_2.fastq.gz, K46_31147_TGACCA_2.fastq.gz,
K50_31148_ACAGTG_2.fastq.gz, K52_31149_GCCAAT_2.fastq.gz
S3K_1_07204AAB_AGGATC_2.fastq.gz, S3K_1_07204AAB_AGGATC.fastq.gz, S3K_2_07205AAB_AACGGT_2.fastq.gz,
S3K_2_07205AAB_AACGGT.fastq.gz, S3K_3_07206AAB_CAGCGT_2.fastq.gz, S3K_3_07206AAB_CAGCGT.fastq.gz,
S3W_1_07202AAB_TGACTT_2.fastq.gz, S3W_1_07202AAB_TGACTT.fastq.gz, S3W_2_07203AAB_TAAGAT_2.fastq.gz,
S3W_2_07203AAB_TAAGAT.fastq.gz, S1K_1_07199AAB_GATGGT_2.fastq.gz, S1K_1_07199AAB_GATGGT.fastq.gz,
S1K_2_07200AAB_AGAGGT_2.fastq.gz, S1K_2_07200AAB_AGAGGT.fastq.gz, S1K_3_07201AAB_CTTCCA_2.fastq.gz,
S1K_3_07201AAB_CTTCCA.fastq.gz, S1W_1_07196AAB_GAACCG_2.fastq.gz, S1W_1_07196AAB_GAACCG.fastq.gz,
S1W_2_07197AAB_CTAAGC_2.fastq.gz, S1W_2_07197AAB_CTAAGC.fastq.gz, S1W_3_07198AAB_AATGCA_2.fastq.gz,
S1W_3_07198AAB_AATGCA.fastq.gz, KO1AC_07490AAC_ATGTCA_R1_001_2.fastq.gz,
KO1AC_07490AAC_ATGTCA_R1_001.fastq.gz, KO1M3_07495AAC_CACCGG_R1_001_2.fastq.gz,
KO1M3_07495AAC_CACCGG_R1_001.fastq.gz, KO2AC_07491AAC_GTTTCG_R1_001_2.fastq.gz,
KO2AC_07491AAC_GTTTCG_R1_001.fastq.gz, KO2M3_07496AAC_CCAACA_R1_001_2.fastq.gz,

KO2M3_07496AAC_CCAACA_R1_001.fastq.gz, KO3AC_07492AAC_GGTAGC_R1_001_2.fastq.gz, KO3AC_07492AAC_GGTAGC_R1_001.fastq.gz, WT1AC_07487AAC_TTAGGC_R1_001_2.fastq.gz, WT1AC_07487AAC_TTAGGC_R1_001.fastq.gz, WT1M3_07493AAC_ACTGAT_R1_001_2.fastq.gz, WT1M3_07493AAC_ACTGAT_R1_001.fastq.gz, WT2AC_07488AAC_TAGCTT_R1_001_2.fastq.gz, WT2AC_07488AAC_TAGCTT_R1_001.fastq.gz, WT2M3_07494AAC_CAACTA_R1_001_2.fastq.gz, WT2M3_07494AAC_CAACTA_R1_001.fastq.gz, WT3AC_07489AAC_GGCTAC_R1_001_2.fastq.gz, WT3AC_07489AAC_GGCTAC_R1_001.fastq.gz1A_2_07497AAC_TCGAAG_R1_001_2.fastq.gz, 1A_2_07497AAC_TCGAAG_R1_001.fastq.gz, Kdm1a_07213AAB_CGCAGG_2.fastq.gz, Kdm1a_07213AAB_CGCAGG.fastq.gz LMH0019_GT18-14688_TCCTGAGC-GCGTAAGA_S2_R1_001.fastq.gz, LMH0019_GT18-14688_TCCTGAGC-GCGTAAGA_S2_R2_001.fastq.gz, LMH0019H_GT19-03422_TCCTGAGC-TCTTACGC_S9_L008_R1_001.fastq.gz, LMH0019H_GT19-03422_TCCTGAGC-TCTTACGC_S9_L008_R2_001.fastq.gz, LMH0019N_GT19-04065_TCCTGAGC-TCTTACGC_S1_R1_001.fastq.gz, LMH0019N_GT19-04065_TCCTGAGC-TCTTACGC_S1_R2_001.fastq.gz 1WT_lib06246AAD_ATCACG_R1_0001.fastq.gz, 2WT_lib06247AAD_CGATGT_R1_0001.fastq.gz, 3KO_lib06248AAD_TTAGGC_R1_0001.fastq.gz, 4WT_lib06249AAD_ATCACG_R1_0001.fastq.gz, 5WT_lib06250AAD_CGATGT_R1_0001.fastq.gz, 6KO_lib06246AAD_TGACCA_R1_0001.fastq.gz, 8KO_lib06252AAD_GCCAAT_R1_0001.fastq.gz

Genome browser session (e.g. UCSC)

No longer applicable

Methodology

Replicates

The number of replicates varies among the different NGS techniques. In the case of ChIP-seq experiments, we considered at least two-three experimental replicates per group. RNAseq was performed in triplicates. For ChIA-pet we just performed 1 replicate. For 4C-seq triplicates were done in two different batches that were corrected during the analysis.

Sequencing depth

RNA-seq and ChIP-seq samples were sequenced in Illumina HiSeq2500 in a single-end 50 bp length configuration. ChIAPETwas sequenced in Miseq, Nextseq 500/550 and Hiseq 3000/4000 in paried-end 150 pb configuration. For 4C-seq was sequenced in Novaseq and were all trimmed to 75pb.

Antibodies

The primary antibodies used were α-Kdm1a (ab17721, 1:200 or 1/1000), a-Histone H3 (tri methyl K4) from Millipore (07-473), a-Histone H3 (mono methyl K4) from Abcam (ab8895), α-H3K27me3 (ab6002, Abcam, 1:250) and α-H3K27ac (ab4729, Abcam, 1:500).

Peak calling parameters

Reads were mapped with Bowtie2 to mm10 genome index. Peak calling was done with MACS2 with default parameters including the input sample.

Data quality

Adapters were trimmed using TrimGalore v 0.6.4_dev, and the reads obtained were mapped to the mouse genome (mm10) with Bowtie2 (v2.3.4.3). Only those reads with a mapq > 30 were selected.
For Kdm1a peaks, IDR algorithm was used, and we selected those that presented a scale higher than IDR value 955. For differential binding analysis we considered just those peaks that were at least present in 2 samples within the same genotype.

Software

Samtools v1.10 ; FastQC v0.11.9 ;Bedtools v2.27.1; DeepTools v3.5.0; DESeq2 v1.26.0; TrimGalore v0.6.4_dev; Rsubread v. 2.4.3; Bowtie2 v2.3.4.3; macs2 v2.1.1.20160309; idr v2.0.2; Diffbind v 2.10.0; ChipPeakAnno v3.24.2; Biomart v2.46.3; homer v4.11.1.

Flow Cytometry

Plots

Confirm that:

☒ The axis labels state the marker and fluorochrome used (e.g. CD4-FITC).

☒ The axis scales are clearly visible. Include numbers along axes only for bottom left plot of group (a 'group' is an analysis of identical markers).

☒ All plots are contour plots with outliers or pseudocolor plots.

☒ A numerical value for number of cells or percentage (with statistics) is provided.

Methodology

Sample preparation

Mice were sacrified by cervical dislocation, hippocampi were microdissected and washed in PBS. Nuclei were extracted after tissue disruption with a dounce homogenizer.

Instrument

FACS Aria III

Software

Equipment software for FACS Aria III

Cell population abundance

The purity of the post-sorted fractions were 98-99% as determined by re-sorting of sorted nuclei and observation in a neubauer chamber.

Gating strategy	For the ChiAPET experiment nuclei samples were first gated by their DAPI signal. Subsequently the singlets were preselected by their FSC-H to FSC-A ratio and SSC-A to SSC-H ratio (see representative example in Supplementary Figure 4H). Finally, nuclei were sorted by their positive fluorescent signal of interest (GFP). Population boundaries were clearly separated.

☒ Tick this box to confirm that a figure exemplifying the gating strategy is provided in the Supplementary Information.
